# Developing a policy brief on physical activity promotion for children and adolescents

**DOI:** 10.3389/fpubh.2023.1215746

**Published:** 2023-09-22

**Authors:** Sven Messing, Peter Gelius, Karim Abu-Omar, Isabel Marzi, Franziska Beck, Wolfgang Geidl, Eva Grüne, Antonina Tcymbal, Anne Kerstin Reimers, Klaus Pfeifer

**Affiliations:** ^1^Department of Sport Science and Sport, Friedrich-Alexander-Universität Erlangen-Nürnberg, Erlangen, Germany; ^2^Institute of Sport Sciences, Université de Lausanne, Lausanne, Switzerland

**Keywords:** physical activity promotion, policy brief, policy consultation, children, adolescents, recommendations, projects, policy

## Abstract

**Introduction:**

While there are several approaches to collect basic information on physical activity (PA) promotion policies, some governments require more in-depth overviews on the situation in their country. In Germany, the Federal Ministry of Health expressed its interest in collecting detailed data on target group specific PA promotion, as relevant competences are distributed across a wide range of political levels and sectors. This study describes the development of a policy brief on physical activity promotion for children and adolescents in Germany. In particular, it addresses two major gaps in the current literature by systematically assessing good practice examples and “routine practices,” i.e., PA promotion activities already taking place on large scale and regular basis.

**Materials and methods:**

Based on relevant national and international guidelines, the TARGET:PA tool was co-produced by researchers and ministry officials. It includes (1) PA recommendations, (2) national prevalence rates, (3) recommendations for PA promotion, and data on national (4) routine practices, (5) good practice projects and (6) policies. Data were collected for children and adolescents in Germany using desk research, semi-structured interviews and secondary data analysis.

**Results:**

A policy brief and scientific background document were developed. Results showed that 46% of the 4–5-year-olds fulfil WHO recommendations but only 15% of the 11–17-year-olds, and that girls are less active than boys. Currently, in Germany no valid data are available on the PA behaviour of children under the age of three. An overview of routine practices for PA promotion for children and adolescents was compiled, and experts were asked to critically assess their effectiveness, reach and durability. Overall, 339 target group specific projects for PA promotion were found, with 22 classified as examples of good practice. National PA policies for children and adolescents were identified across different sectors and settings.

**Conclusion:**

The study provides a comprehensive overview of the current status of PA promotion for children and adolescents in Germany. The co-production of the policy brief was a strength of the study, as it allowed researchers to take the needs of ministry officials into account, and as it supported the immediate uptake of results in the policymaking process. Future studies should test the applicability of the TARGET:PA tool to different target groups and countries.

## Introduction

Physical activity (PA) is a key determinant for the health of children and adolescents, with a positive influence on cardiovascular health, motor fitness, and body weight (1, 2). In addition, regular PA supports physical and mental development (3) and academic performance (4). As an active lifestyle at a young age shapes PA behaviour later in life, promoting PA in children and adolescents is also an investment in the future health of the population (5, 6).

However, while there is sufficient evidence of the health effects of PA, 81% of adolescents aged 11–17 years do not meet the recommendations of the World Health Organization (WHO) for 60 min of moderate- to high-intensity PA per day (7, 8). For this reason, promoting PA within this target group is of outstanding importance, and the effectiveness of respective interventions and policies has been shown in the scientific literature (9–11). Furthermore, international policy documents such as WHO’s Global Action Plan for Physical Activity include specific recommendations for promoting PA among children and adolescents to guide national policy development (12).

To inform the development and review of target group-specific policies to promote PA for children and adolescents, an in-depth analysis of the current status of PA promotion within a country is an important step. For this reason, the German Federal Ministry of Health initiated a data collection exercise including prevalence rates of children and adolescents, target group-specific routine practices, projects, and policies for PA promotion. An important reason for developing this policy brief were consistently high levels of physical inactivity among children and adolescents in Germany, which were exacerbated by the COVID-19 pandemic due to the closure of day care centers, schools and sport facilities for extended periods of time (13). In addition, children and adolescents are an important target group in the update of the National Action Plan “IN FORM - Germany’s Initiative for Healthy Nutrition and More Physical Activity” (14, 15).

Compared to previous initiatives to monitor PA behaviour and PA promotion practices (16–18), the policy brief addresses two major knowledge gaps: First, rather than following established practice by identifying good practice projects based on expert assessment, it employed an objective and systematic process to ensure the selection of high-quality projects that could be proposed for future scale-up. Second, “routine practices” tend to be a blind spot of current monitoring initiatives, i.e., PA promotion activities taking place on large scale and regular basis. The study at hand systematically assessed such routine practices in Germany, as they are particularly relevant to policymakers due to their high reach and potential public health impact (19).

This manuscript aims to describe the current status of PA promotion for children and adolescents in Germany. It also reflects on the first application of the newly developed data collection tool (TARGET:PA tool; see reference 19) and discusses the added value of this study compared to other attempts to monitor PA behaviour, routine practices, and policies at the national level.

## Materials and methods

**Figure 1:**
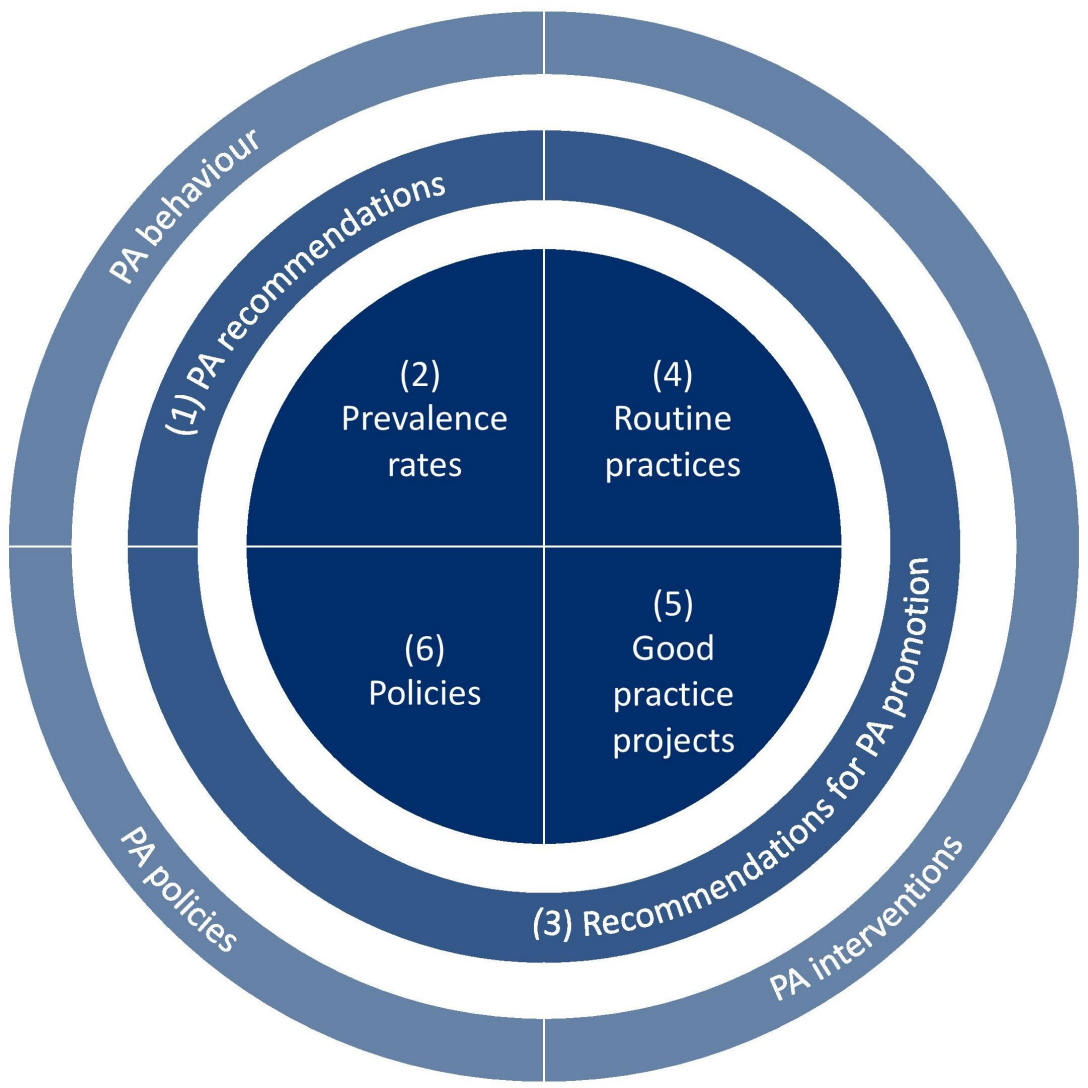
The TARGET:PA tool.

Data were collected from March to August 2021 using the newly developed TARGET:PA tool that is based on the typology of three types of scientific evidence: PA behaviour, PA interventions, and PA policies (20). In addition, the tool is aligned with two groups of recommendations: PA recommendations that are targeted at individuals (recommended nature, duration, intensity, and volume of PA) and recommendations for PA promotion that target governments and stakeholders (interventions and policies for PA promotion) (21). The tool includes six elements (1): PA recommendations (2), prevalence rates (3), recommendations for PA promotion (4), practices that take place on a routine basis, e.g., due to legal regulations, funding mechanisms or the initiative of organisations (5), evidence-based projects that have proven their efficacy, and (6) policies (Figure 1).

### PA recommendations

A comparison and synthesis of Germany’s National Recommendations for PA and PA Promotion (22) with WHO’s Guidelines on PA and Sedentary Behaviour (8) and WHO’s Guidelines for Children under 5 years of age (23) was performed. WHO’s previous PA guidelines were also included in this comparison (24), as these recommendations were used as a threshold in several studies of prevalence rates (section 2).

### Prevalence rates

Data on PA prevalence rates of children and adolescents in Germany were collected in a four-step process. First, relevant studies that were collected in three scientific databases (Web of Science, Pubmed, Scopus) were received from the German Active Healthy Kids Network. In addition, a systematic search was conducted to double-check and complement the results. Next, researchers sorted the studies by age (0–2 years, 3–5 years, 6–10 years, 11–17 years) and type of PA behaviour (total PA, organised sports, unorganised sport/active play, PA at a childcare facility/school, sedentary behaviour, active transport). Data on the adherence to PA recommendations were extracted for different age groups; gender-specific differences, socio-economic inequalities, and the changes of PA behaviour during the COVID-19 pandemic were also analysed. In addition, data on the sample size were extracted for each study (per age group / PA behaviour) and a bubble chart was created to visualize differences in data availability.

### Recommendations for PA promotion

A synthesis of recommendations for PA promotion was performed based on five national, European, and global documents: (1) Germany’s National Recommendations for PA and PA Promotion (22), (2) WHO’s Global Action Plan for PA (12), (3) PA Strategy for the WHO European Region 2016–2025 (25), (4) Council Recommendation on promoting Health-Enhancing PA across sectors (26), and (5) the International Society for PA and Health’ Eight Investments that work for PA (27).

To structure the synthesis, categories were developed based on the sectors/settings targeted by the recommendations. These categories were also used to structure the data on routine practices, projects, and policies (see section 4–6).

### Routine practices

To identify routine practices, semi-structured expert interviews were conducted. In order to identify experts, the research team created a comprehensive list of 46 individuals with a high expertise in research and practice, covering all categories with recommendations for PA promotion (see section 3). The suitability of the experts was rated on a five-point scale, and for each category the individual with the highest rating was contacted. Six expert interviews were conducted (one expert per category, if possible). These expert interviews took place between April and June 2021 and lasted approximately 45 to 60 min. Experts were asked to identify practices that take place on a routine basis, e.g., due to legal regulations, funding mechanisms or the initiative of organisations (‘routine practices’). For each routine practice, experts were asked to assess the reach, durability, and effectiveness. After each interview, key results were extracted and summarized.

### Good practice projects

To identify evidence-based projects that have proven their efficacy, a systematic search was conducted in national project databases. Databases were identified via a study related to the development of Germany’s National Recommendations for PA and PA promotion (28). Five out of eight databases were still available and included projects targeting children and adolescents (29–33). In a subsequent search for relevant databases, no additional databases were identified.

In order to assess these projects, established quality criteria for the conceptualization, implementation, and evaluation of interventions were applied (34). These quality criteria were structured according to the RE-AIM framework (35); afterwards, their number was reduced based on (a) the measurability of each criterion, (b) the relevance of the criterion for the study, and (c) a combination of related criteria. The following combined quality criteria were identified as being of particular relevance for assessing the identified projects:

- Effectiveness: The project has a theoretical foundation, its outcomes were evaluated, and ideally a cost/benefit ratio was determined.
- Reach: The target group was identified, and the target group reach was evaluated.
- Maintenance: The maintenance of the project is prepared, e.g., by the empowerment of stakeholders, the capacity building of organizations and the structural embeddedness of the project.

Good practice projects were selected and assessed in a four-step process. First, projects were sorted into the previously developed categories (see section 3). Second, data were extracted from project databases. Third, projects were selected as examples of good practice when evidence of their effectiveness was identified (inclusion criterion). Fourth, projects were assessed and described based on the three criteria of effectiveness (project outcome), reach (number of children and adolescents or number of facilities), and maintenance (duration of the project). For assessing and describing the projects, additional sources such as project reports, scientific publications, and project websites were used.

### Policies

Data on policies for PA promotion for children and adolescents were collected via WHO’s Health Enhancing Physical Activity (HEPA) Policy Audit Tools (PAT) (36). These data were obtained from a study within the Policy Evaluation Network (17). As the HEPA PAT is not a target group-specific tool, the results were analysed for policies targeting children and adolescents using (1) a content analysis of HEPA PAT policy documents was conducted to identify links to PA promotion for children and adolescents; additional data were added based on information collected by the WHO Regional Office for Europe as part of the EU HEPA Monitoring Framework (37) and (2) relevant organisations for PA promotion for children and adolescents were identified based on desk research and the results of a study on relevant actors and structures for PA promotion in Germany (38). All results were structured based on the categories developed in section 3.

## Results

### PA recommendations

National and international recommendations for children and adolescents differ slightly with regards to the recommended levels of moderate-to-vigorous physical activity (MVPA) (8, 22–24) (Table 1).

**Table 1:** Synopsis of PA recommendations.

Age	German recommendations	WHO 2010	WHO 2019/2020
0	As much as possible	–	Several times a day
1			At least 180 min/day
2			
3			At least 180 min/day
4	At least 180 min/day		
5		At least 60 min/day	At least 60 min/day
6			
7	At least 90 min/day		
8			
9			
10			
11			
12	At least 90 min/day		
13			
14			
15			
16			
17			
18			

### Prevalence rates

**Figure 2:**
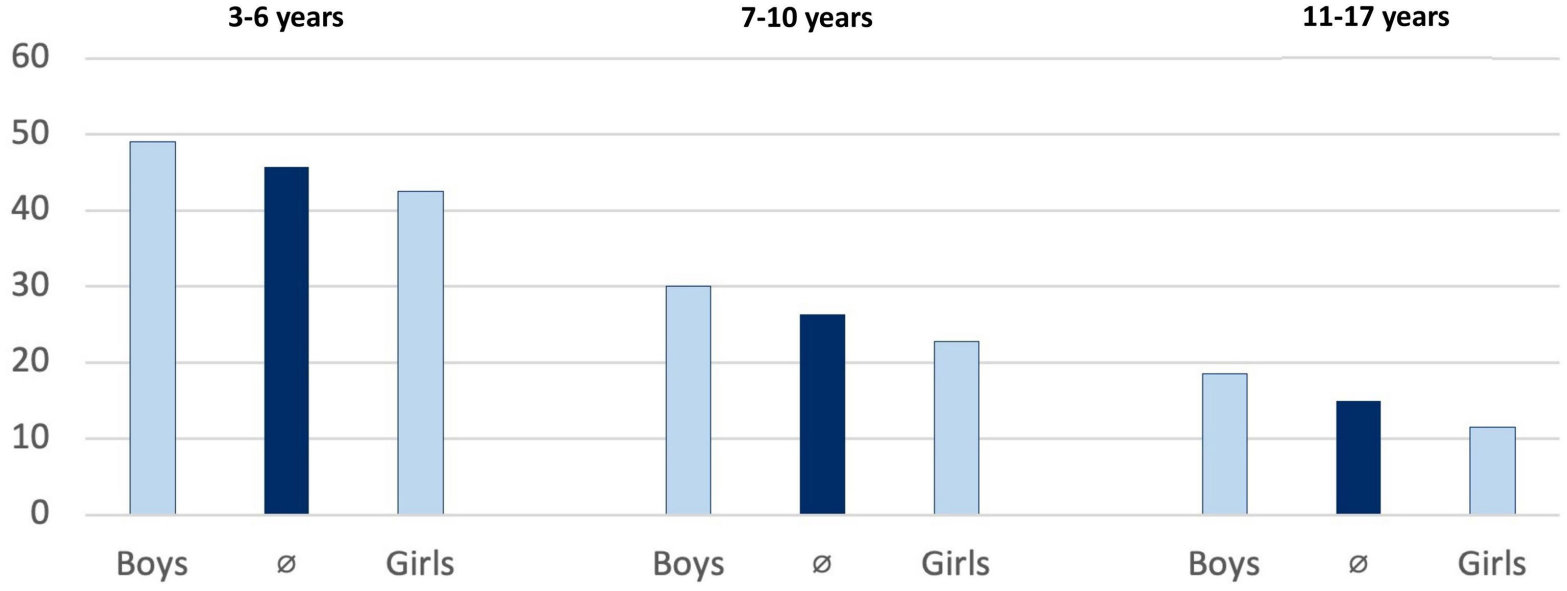
Adherence to WHO recommendations by age and gender (in %).

The representative “German Health Interview and Examination Survey for Children and Adolescents” (KiGGS) showed that 46 percent of 3- to 6-year-olds met the WHO recommendations in 2014–17, but only 15 percent of 11- to 17-year-olds [(39), Figure 2]. The study showed that girls are less active than boys in all age groups, particularly in adolescence. An additional secondary data analysis of included studies confirmed clear gender differences, showed that the PA behaviour of children and adolescents in Germany depends on the socioeconomic status of their parents, and indicated that the COVID-19 pandemic and containment measures had a negative impact on PA levels of children and adolescents (40).

Most data are available for adolescents aged 11 to 17 years, compared to younger age groups. Currently, there is no data available on the PA behaviour of 0- to 2-year-olds in Germany, and data for children aged 3 to 5 years is limited. Most studies collected data on overall PA levels or the participation in organized sports. Data availability on active transport, active play, PA at childcare facilities or schools, and sedentary behaviour is limited (Table 2).

**Table 2:** Data availability on PA prevalence rates of children and adolescents in Germany (size of the circle is proportional to the sample size of the study).

	0–2 years	3–5 years	6–10 years	11–17 years
Total physical activity		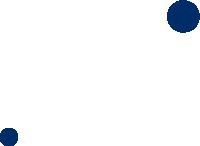	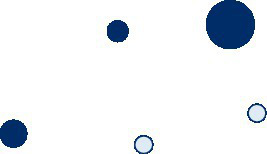	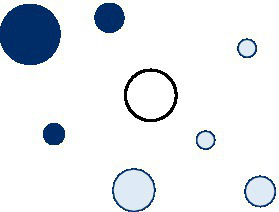
Organized sports		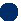	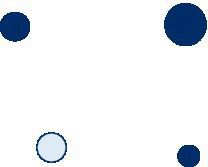	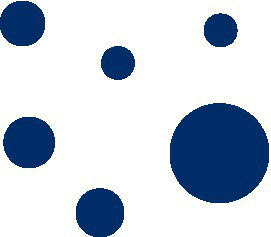
Unorganized sports / active play		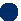	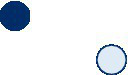	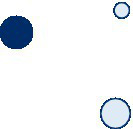
Physical activity at childcare facility / school		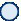	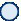	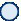
Sedentary behaviour			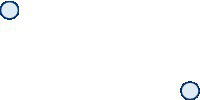	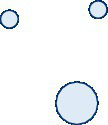
Active transport to and from childcare facility / school		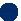	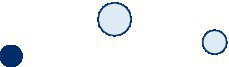	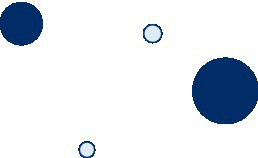

The colour of the circle describes the study type (white: international studies; dark blue: national representative study; light blue: regional/local study).

### Recommendations for PA promotion

Recommendations for PA promotion exist for the following settings and sectors (1): family and home (2), childcare (3), school (4), sport (5), health (6), transport (7), urban planning, and (8) other (12, 22, 25–27). Most recommendations focus on the school and childcare setting. Some of the recommendations for other settings and sectors are directly targeting children and adolescents (e.g., promotion of active transport to and from schools), while others are relevant for all age groups (e.g., creating compact cities; Table 3).

**Table 3:** Synopsis of national and international recommendations for PA promotion for children and adolescents.

	Recommendations for PA promotion
Family and home setting	Provide information to future parents and young families about the importance of PA during pregnancy and for small children Provide and ensure access to facilities and schemes for pregnant women and parents with infants and young children to be active Implement programmes aimed at families, parents, and caregivers to develop the necessary skills to help young children enjoy active play and explore within the family environment Actively involve parents in PA promotion interventions for their children
Childcare	Create a physical activity promoting environment Implement programmes for physical activity promotion nationwide Implement guidance for physical activity promotion (including guidance on facility design) Qualify childcare professionals Ensure the availability of appropriate teaching resources and materials Use regulation or fiscal measures to promote the inclusion of children from vulnerable groups and children with disabilities Involve parents actively in PA promotion interventions for their children
School	Provide regular, high quality physical education lessons Increase the amount of time spent on physical activity and the quality of physical activity programmes Implement school-related physical activity promotion programmes Implement a whole-of-school approach / multicomponent approach for physical activity promotion at schools Create a physical activity promoting environment Include physical activity promotion in school curricula Include physical activity promotion in the training curricula and professional development of all teachers and provide appropriate teaching resources and materials Promote the inclusion of children from vulnerable groups and children with disabilities Involve parents actively in PA promotion interventions for their children Establish appropriate monitoring mechanisms
Sport	Open up existing indoor and outdoor spaces for physical activity (e.g., sports halls, school yards) Promote the participation of children and adolescents in out-of-school physical activity programmes and support membership of sports and fitness clubs/gyms Increase the access to recreational and sport facilities for children from socially disadvantaged groups Adopt a national sport for all policy and/or action plan Implement the health-oriented sport clubs’ guidelines (Sport Clubs for Health Programme) Support scientific research on physical activity promotion by sport clubs
Health	Implement counselling on physical activity performed by health professionals, also to future parents Implement training on physical activity into the curriculum for health professionals Ensure monitoring and surveillance of physical activity and sedentary behaviour
Transport	Promote active transport of children and adolescents to and from schools Promote walking and cycling to school and offer cycling and road safety training Adopt provisions for safe active commuting to childcare facilities and schools
Urban Planning	Create compact cities that locate shops, schools, other services, parks and recreational facilities, as well as jobs near homes, and provide a highly connected street network for walking and cycling Apply the European Guidelines for improving Infrastructures for Leisure-Time Physical Activity systematically
Other	Promote physical activity through intersectoral approaches of the health, sport and education sector

### Routine practices

Routine practices for PA promotion for children and adolescents that take place on a regular basis were identified for most of the categories identified in section 3, except urban planning and other (Table 4).

**Table 4:** Routine practices, good practice projects, and policies for physical activity promotion for children and adolescents in Germany.

	Routine practices	Good practice projects	Policies
Family and home setting	Informative leaflets for medical check-ups Parent–child gymnastics Access to physical activity programs in settings (childcare, day care) Childcare facilities as family centres Lay multipliers for physical activity promotion	–	–
Childcare	Gymnastic lessons in facilities Free play in the gym in the morning Going outside every day Forest days Cooperation with sport clubs and other organisations Training of childcare professionals	TigerKids-Kindergarten aktiv; Nürnberger Netzwerk Bewegungspädagogik; Pfiffikus durch Bewegungsfluss; LOTT-JONN Initiative Kinder- und Jugendgesundheit; KIKS UP KLASSE KLASSE; Fit zur Schule; Teilprojekt von SMS: ‘Fitness für Kids’; Hüpfdötzchen - Kindergarten in Bewegung; JolinchenKids - Fit und gesund in der Kita	Gym / physical activity room as a formal requirement for the registration of childcare facilities Physical activity part of the training curriculum of childcare professionals
School	Physical education Extracurricular physical education offers or competitions Active breaks Active lessons Hiking days School trips (‚Schullandheim‘) Project weeks / days	DIE RAKUNS – das gesunde Klassenzimmer; Bewegte Ganztagsschule; Fit4future; JuvenTUM; Futbalo Girls; ScienceKids: Kinder entdecken Gesundheit; Fit durch die Schule; Klasse2000; Gesund macht Schule; Komm mit in das gesunde Boot; Klasse in Sport; Schulkids in Bewegung – Meine Schule, mein Verein	3–6 obligatory lessons of physical education per week at primary and secondary schools Physical activity and health part of the teacher training curriculum
Sport	Sport club programmes Cooperation with schools and childcare facilities Cooperation with other organisations	–	–
Health	Medical check-ups (including materials)	–	Physical activity counselling obligatory component of medical check-ups (‘U-Untersuchungen’) Medical counselling includes – if needed – information on regional support services for parents and children Entitlement of children and adolescents to check-ups that include – if needed – a recommendation on prevention
Transport	Walking Bus (to and from schools) Traffic-calmed zones around schools Parking spaces close to schools but not directly in front of the building Expansion of walking and cycling paths Transport development plans (in some cases also local mobility concepts or master plans for playing)	The Daily Mile	–
Urban Planning	–	–	Regulations on the accessibility of urban play areas (DIN 18034: 2012–09)
Other	–	–	Health promotion and prevention in settings (childcare facilities, school, children’s and youth facilities)

In total, 27 routine practices were identified. According to the interviewed experts, the durability of the majority of routine practices was considered to be high (63.0%). However, for less than half of the practices, only the effectiveness (48.1%) and reach (40.7%) were considered to be high. Examples for routine practices with a high reach, durability, and effectiveness were identified in the school sector (school trips) and education sector (traffic-calmed zones around schools, expansion of walking and cycling paths, and transport development plans; Table 5).

**Table 5:** Assessment of routine practices for physical activity promotion for children and adolescents in Germany.

	Routine practices	Estimated reach	Estimated durability	Estimated effectiveness
Family and home setting	Informative leaflets for medical check-ups	Medium	High	High
	Parent–child gymnastics	Medium	High	Low
	Access to physical activity programs in settings (childcare, day care)	Medium	N/A	N/A
	Childcare facilities as family centres	Low	N/A	N/A
	Lay multipliers for physical activity promotion	Low	N/A	High
Childcare	Gymnastic lessons in childcare facilities	High	High	Medium
	Free play in the gym in the morning	Medium	High	Low
	Going outside every day	Medium	High	Medium
	Forest days	Medium	Medium	Medium
	Cooperation with sport clubs and other organisations	Low	High	High
	Training of childcare professionals	High	High	Medium
School	Physical education	High	High	Medium
	Extracurricular physical education offers or competitions	Low	N/A	High
	Active breaks	High	High	Medium
	Active lessons	Low	High	High
	Hiking days	High	High	Low
	School trips (‘Schullandheim’)	High	High	High
	Project weeks / days	Low	N/A	High
Sport	Sport club programmes	Medium	High	High
	Cooperation with schools and childcare facilities	Low	High	High
	Cooperation with other organisations	Low	N/A	N/A
Health	Medical check-ups (including materials)	High	Low	Low
Transport	Walking Bus (to and from schools)	High	Low	High
	Traffic-calmed zones around schools	High	High	High
	Parking spaces close to schools but not directly in front of the building	N/A	Medium	N/A
	Expansion of walking and cycling paths	High	High	High
	Transport development plans (in some cases also local mobility concepts or master plans for playing)	High	High	High
Urban Planning	-	-	-	-
Other	-	-	-	-

### Good practice projects

The database search resulted in 339 projects on PA promotion for children and adolescents. After excluding duplicates and irrelevant projects, the 155 remaining projects were sorted into the eight categories. The majority of projects (65%) took place in childcare facilities or schools. Twenty-two projects met the inclusion criteria and were classified as good practice projects due to their proven effectiveness and a promising reach and/or duration (Table 3).

The included projects differed with regards to the proven effects, e.g., increase of daily amount of PA (DIE RAKUNS), reduced prevalence of obesity and overweight (TigerKids), or improvement of motor skills (LOTT-JONN). One project reached more than 1,000,000 children in Germany (Klasse2000), four projects between 100,000 and 999,999 participants (JolinchenKids, TigerKids, DIE RAKUNS, fit4future), and five projects between 10,000 and 99,999 participants (Fitness für Kids, Futbalo Girls, Gesund macht Schule, Fit durch die Schule, Komm mit in das gesunde Boot). The remaining 12 projects reached either less than 10,000 children and adolescents (e.g., in pilot studies) or only provided information on the number of classes, schools, or childcare facilities that were reached. One project has been running since the 1990s (Klasse 2000), 13 projects since the 2000s, and five projects since the 2010s. Three projects have already been completed (Hüpfdötzchen, Fit zur Schule, JuvenTUM; Table 6).

**Table 6:** Assessment of good practice projects for physical activity promotion for children and adolescents in Germany.

	Good practice projects	Reach	Durability	Effectiveness
Family and home setting	–	–	–	–
Childcare	KIKS UP	Approximately 140 schools	Since 2004	Positive effects on the assessment of personal fitness and enjoyment of exercise
	‚Fitness für Kids‘– Frühprävention im Kindergarten- und Grundschulalter	More than 1,000 childcare facilities and elementary schools, 15,000 children	Since 2002	Positive effects on motor development and diastolic blood pressure
	JolinchenKids – Fit und gesund in der Kita	121,000 families	Since 2014	Increase of moderate-to-vigorous PA and decrease of sedentary behaviour
	TigerKids – Kindergarten aktiv	5,500 childcare facilities,300,000 families	Since 2005	Positive effects on the prevalence of obesity and overweight
	Hüpfdötzchen – Kindergarten in Bewegung	489 children in pilot study, no additional data available	1996–2013	Improvement of motor skills, increase of physical activity promotion measures in the childcare facility
	Fit zur Schule	97 children in pilot study	2014–2017	Reduced proportion of children with difficulties related to speaking, gross motor skills, and perception
	LOTT-JONN Initiative Kinder- und Jugendgesundheit	More than 170 facilities	Since 2000	Improvement of motor skills
	Nürnberger Netzwerk Bewegungspädagogik	3,447 children, 63 childcare facilities	Since 2007	Improvement of PA opportunities, increasing knowledge of childcare professionals, improvement of coordination skills, concentration, self-confidence, and problem solving
	Pfiffikus durch Bewegungsfluss	No data available	Since 2002	Improvement of motor skills, positive development of body motor skills
School	Futbalo Girls	40,000 participants	Since 2006	Positive effects on club membership, long-term commitment to the programme, and increased interest in soccer, self-confidence, teamwork, and health awareness
	DIE RAKUNS – das gesunde Klassenzimmer	500,000 children	Since 2013	Increase of daily amount of PA, increase of knowledge about PA
	Klasse2000	1,800,000 participants	Since 1991	Positive effects on active transport to and from schools
	Gesund macht Schule	35,000 participants	Since 2013	Positive effect on coordination and endurance skills
	fit4future	600,000 participants	Since 2005	Increase of regular PA, increase of performance in the shuttle run test and single leg stand, improvement of visuomotor coordination
	Klasse in Sport – Initiative für täglichen Schulsport e.V.	Approximately 100 schools	Since 2006	Positive effects on sport motor skills, BMI, and sport club activity, positive effects on social behaviour and interest in sport
	ScienceKids: Kinder entdecken Gesundheit	37 schools in pilot study	Since 2007	Positive effects on PA outside school and sport with the family, increase in competence/knowledge of PA
	Fit durch die Schule	20,000 participants (814 projects)	Since 2009	Increase of enjoyment of PA, sport, and physical education, increase in participation in extracurricular sport, positive effects on fitness
	Komm mit in das gesunde Boot	90,000 children, parents and childcare professionals	Since 2006	Positive effects of endurance capacity, mobility, and the development of obesity, tendency to increase moderate-to-vigorous PA
	Bewegte Ganztagsschule	3,000 children, 7 schools	Since 2008	Positive effects on prevalence of overweight and sport club activity, improvement in endurance performance
	JuvenTUM	32 classes	2007–2014	Tendency to improve overall PA and physical fitness, reduction of abdominal girth
	Schulkids in Bewegung – Meine Schule, mein Verein	4,600 children, 34 schools	Since 2011	Positive effects on motor and sport-related performance (especially for children with a migration background)
Sport	–	–	–	–
Health	–	–	–	–
Transport	The Daily Mile	3,000,000 children (international), 45 schools in Germany	Since 2012	Increase in overall moderate-to-vigorous PA, reduction in sedentary behaviour, improvement in shuttle run test, positive effect on body composition
Urban Planning	–	–	–	–
Other	–	–	–	–

### Policies

Specific regulations to promote PA for children and adolescents exist in different settings and sectors (see examples in Table 3). Additionally, at the level of the federal states, a regular monitoring of physical education lessons takes place and different PA promotion programmes are in place (e.g., “active school,” “walking bus”). Furthermore, the education sector invites representatives of the sport and health sector to participate in the development of the physical education curriculum. In the urban planning sector, single programmes such as “Social City” (*Soziale Stadt*) or “Experimental Housing and Urban Development” (*Experimenteller Stadt- und Wohnungsbau*) are linked to health promotion.

Besides these very specific regulations, a number of key policy documents for PA promotion include policies for children and adolescents, especially for the childcare and school setting (Table 7).

**Table 7:** National policy documents for PA promotion for children and adolescents.

	National action plan IN FORM 2008	National recommendations for PA and PA promotion 2016	National basic recommendations of the national preventive conference 2018	Guidance document for prevention of the statutory health insurances 2020	National cycling plan 3.0 2021
Family and home setting	X	X	X		
Childcare	X	X	X	X	X
School	X	X	X	X	X
Sport				X	
Health	X				
Transport					X
Urban planning	X				
Other					

## Discussion

This study used the new TARGET:PA tool to provide a comprehensive overview of the current status of PA promotion for children and adolescents in Germany. Results showed that 46% of the 3- to 6-year-olds and 15% of the 11- to 17-year-olds fulfil WHO recommendations, and that girls are less active than boys. Currently, no valid data are available on the PA behaviour of 0- to 2-year-olds in Germany. An overview of routine practices for PA promotion for children and adolescents was compiled, and experts were asked to critically assess their effectiveness, reach, and durability. Overall, 339 target group specific projects for PA promotion were found, with 22 classified as good practice projects. National PA policies for children and adolescents were identified across different sectors and settings.

An innovative aspect of this study is the identification of gaps in data availability. Besides the lack of valid data on the PA prevalence rates of 0- to 2-year-olds, data on the PA behaviour of 3- to 5-year-olds were also limited. The analysis also showed that the studies with the largest sample sizes were conducted among the oldest age group (11- to 17-year-olds), indicating that data availability improves for higher aged children and adolescents. These age differences in data availability might be caused by methodological difficulties related to the measurement of PA in infants and young children. While data on participation in organized sports were collected in several surveys, data availability on active transport and PA behaviour at school – outside physical education lessons – is limited. There are also limited studies and inconclusive data on the influence of the parents‘socioeconomic status and migration background on the PA behaviour of their children as well as on gender-dependent social gradients.

Another novel aspect in this study is the integrated analysis of recommendations for PA promotion, routine practices, good practice projects, and policies. The analysis of these elements was based on eight categories that were derived from national and international recommendations for PA promotion. A strong focus on the childcare and school setting was identified – especially for projects (21 out of 22 good practice projects targeted one of these two settings) but also for recommendations and policies. The inclusion of routine practices is a unique focus of this study; data on this aspect have hardly been collected previously in the field of PA promotion, as research usually focuses either on identifying good practice projects (e.g., reference 28) or on monitoring policies for PA promotion (e.g., reference 41). However, this aspect is especially relevant as the reach of routine practices is often much higher than the reach of single projects. In contrast, in many cases the effectiveness of routine practices for PA promotion has not been investigated, while the selected good practice projects have proven their effectiveness. This calls for analysis of the effectiveness of routine practices and raises the question of how routine practices can be modified to increase their public health impact.

This study of PA promotion for children and adolescents in Germany was the first application of the newly developed TARGET:PA tool (19). Based on this study, researchers and ministry officials co-produced a policy brief (42) and a scientific background document (40). Both documents were published by the Federal Ministry of Health in the context of a national Physical Activity Summit in December 2022 that focused on promoting sports and PA, especially among children and adolescents – as this target group was particularly affected by the COVID-19 pandemic due to the closure of sport facilities and the cancellation of physical education lessons (43). In the context of this summit, the Federal Ministry set up a Round Table on PA and Health with key stakeholders from different sectors that aimed to agree on specific measures for target group-specific PA promotion. The above-mentioned policy brief was an important basis for this Round Table and was also utilized as the first in a series of brief updates to the German National Recommendations for PA and PA promotion conducted under the auspices of the Federal Ministry of Health. As such it had an immediate impact on the political debate of key stakeholders.

This study has several limitations that need to be considered. First, the study was conducted on an *ad hoc* basis, i.e., based on an urgent request from the Ministry of Health and not planned in advance. In order to inform policymaking within and outside the Federal Ministry of Health in a timely manner, data collection was based on a rapid but systematic process. However, if time was not limited, the methodology could have been adjusted to collect more data on specific aspects such as routine practices and/or to analyse data more in-depth. Second, the identification of experts for the semi-structured interviews was difficult for sectors that are relevant for PA promotion but might not necessarily perceive this as one of their key tasks (e.g., urban planning). For this reason, the overview of routine practices might not be complete. However, due to the limited body of evidence on this aspect, this is still a step forward and could be a starting point for future research. Third, the relevant settings for PA promotion were analysed separately and intersectoral initiatives for PA promotion were not identified systematically. Fourth, with regards to the identification of good practice projects, it must be noted that existing databases do not provide a complete overview about all projects for PA promotion. In some cases, project databases seemed to be outdated (i.e., no new projects have been added in recent years) and did not provide access to further information (i.e., websites, project reports). The incentive for a database entry was unclear in many databases and the provided information was very heterogenous. Lastly, data collection on PA promoting policies was based on tools that do not collect target group specific information, such as WHO’s HEPA PAT and the EU/WHO HEPA Monitoring Framework, and is limited to national level policies. Additional target group specific surveys and research focusing on the subnational level could help to identify additional policies relevant for PA promotion of children and adolescents in Germany.

The following key conclusions for policymaking in Germany can be drawn from this study:

- *PA recommendations*: Existing national and international recommendations for children and adolescents vary due to their different years of origin and the advancing scientific evidence. National recommendations should be updated in regular intervals, e.g., every 5 years.
- *Prevalence rates*: Efforts for PA promotion for children and adolescents need to be increased, as a decreasing proportion meet current PA recommendations as they get older. The needs of girls should be given special consideration. In addition to the continuous monitoring of PA behaviour in children and adolescents from (pre-)school age, an initial data collection is needed for children under 3 years of age.
- *Recommendations for PA promotion*: National and international recommendations for PA promotion should be implemented systematically.
- *Routine practices*: The reach and effectiveness of routine practices for PA promotion among children and adolescents should be increased and monitored on a regular basis.
- *Good practice projects*: The nationwide dissemination of good practice projects in the school, childcare facilities, and transport settings should be examined. As no good practice project was identified for other settings, future studies should investigate the effectiveness of projects in these settings.
- *Policy*: A systematic monitoring of policies for PA promotion in Germany should be implemented across the different levels of government (national level, federal states, and municipalities). In addition, the networking of relevant organizations needs to be facilitated across political levels and sectors to strengthen structures for PA promotion in Germany.

## Conclusion

From a more general perspective, the study was the first implementation of the TARGET:PA tool and provided a comprehensive overview of the current status of PA promotion for children and adolescents in Germany. Furthermore, the study confirmed the added value of the tool for monitoring activities in the field of PA promotion, and it closed a research gap by systematically assessing good practice examples as well as routine practices. The co-production of the policy brief and the scientific background document was a strength of the study, as it allowed researchers to take the needs of ministry officials into account at each stage of the process. This supported the immediate uptake of the results in the policymaking process coordinated by the Federal Ministry of Health, e.g., for the establishment of a national and intersectoral Round Table on PA and Health. The TARGET:PA tool is designed to be applicable to other target groups and in other countries; however, future studies need to test whether the tool needs to be modified when applied in another context.

## Data availability statement

The original contributions presented in the study are included in the article/supplementary material, further inquiries can be directed to the corresponding author.

## Ethics statement

Ethical approval was not required for the study involving humans in accordance with the local legislation and institutional requirements. Written informed consent to participate in this study was not required from the participants or the participants’ legal guardians/next of kin in accordance with the national legislation and the institutional requirements.

## Author contributions

All authors contributed to study conceptualization and design. FB, KA-O, and AR analysed data related to PA recommendations and prevalence rates. SM and PG analysed recommendations for PA promotion and policies. KA-O, SM, EG, and WG analysed routine practices. IM analysed good practice projects. PG and SM coordinated data collection and analysis, KP and AR supervised the study. SM wrote the original manuscript draft. KA-O, PG, and AT provided feedback on early drafts. All authors contributed to the article and approved the submitted version.
